# Advances in Engineered Macrophages: A New Frontier in Cancer Immunotherapy

**DOI:** 10.1038/s41419-024-06616-7

**Published:** 2024-04-01

**Authors:** Shuaixi Yang, Yuhang Wang, Jiachi Jia, Yingshuai Fang, Yabing Yang, Weitang Yuan, Junhong Hu

**Affiliations:** Department of Colorectal Surgery, The First Affiliated Hospital of Zhengzhou University, 1 East Jianshe Road, Zhengzhou, 450000 China

**Keywords:** Immunotherapy, Cancer therapy

## Abstract

Macrophages, as pivotal cells within the tumour microenvironment, significantly influence the impact of and reactions to treatments for solid tumours. The rapid evolution of bioengineering technology has revealed the vast potential of engineered macrophages in immunotherapy, disease diagnosis, and tissue engineering. Given this landscape, the goal of harnessing and innovating macrophages as a novel strategy for solid tumour immunotherapy cannot be overstated. The diverse strategies for engineered macrophages in the realm of cancer immunotherapy, encompassing macrophage drug delivery systems, chimeric antigen receptor macrophage therapy, and synergistic treatment approaches involving bacterial outer membrane vesicles and macrophages, are meticulously examined in this review. These methodologies are designed to enhance the therapeutic efficacy of macrophages against solid tumours, particularly those that are drug-resistant and metastatic. Collectively, these immunotherapies are poised to supplement and refine current solid tumour treatment paradigms, thus heralding a new frontier in the fight against malignant tumours.

## Facts

- The paradigm of cancer treatment has transitioned from a focus on the cancer itself to a treatment model centred on the tumour microenvironment.
- Engineered macrophages can serve as carriers for anticancer or nanoparticle drugs.
- CAR-Ms have broad application prospects in the context of immunotherapy for solid tumours.
- A novel approach to cancer immunotherapy involves utilising tumour vaccines derived from bacterial outer membrane vesicles.

## Open questions

- What are the intrinsic mechanisms by which macrophages act as drug-delivery vehicles? What is their therapeutic efficacy?
- CAR-M-cell therapy may be the main therapeutic approach for solid tumours in the future. Is this therapy feasible in clinical trials?
- Can the interaction between OMVs and macrophages enhance their applicability and diversity in the design of tumour vaccines?
- As a vital element within the solid tumour microenvironment, can macrophages serve as the focal point of interdisciplinary collaboration in the future for the advancement of related therapies?

## Introduction

Cancer is a genomic disease in which a considerable number of somatic point mutations accumulate, leading to structural changes during its development and resulting in genomic instability [1, 2]. Cancer tissues, which are mainly composed of a substantial quantity of both neoplastic and nonneoplastic cells, modify the extracellular matrix and thus generate a unique tumour microenvironment (TME) [3, 4]. The TME is defined as an intricate and dense multicellular environment for tumorigenesis that consists of a substantial amount of different cells and cellular components, including multiple types of immune cells, endothelial cells, tumour-associated macrophages (TAMs), and a variety of other tissue-resident cell types [3, 5–7]. These cells act synergistically in tumour progression, invasion, metastasis, and response to immunotherapies [8]. Consequently, cancer treatment has transitioned from a model centred solely on the cancer itself to one focused on the tumour microenvironment, and cancer immunotherapy has consequently come onto the stage to revolutionise cancer treatment. However, its efficacy is still limited in most clinical settings [9, 10]. In recent years, cancer immunotherapy has been pursued through a myriad of approaches, including molecular targeted therapy, immune checkpoint inhibitors (such as PD-1/L1 and CTLA-4 inhibitors), adoptive cell immunotherapy (such as TIL, NK, CAR-T, CIK/DC-CIK), cytokine therapy, and tumour vaccines [2, 11–13].

Macrophages, as heterogeneous and multifunctional immune cells, play crucial roles in various biological processes, such as maintaining tissue homoeostasis, regulating cancer progression, and defending against pathogens. Their phenotype and functionality are intricately governed by the ambient microenvironment, and macrophages can demonstrate dual antitumour and tumour-promoting effects within the context of cancer [14, 15]. Polarised macrophages can be classified into two distinct subtypes, M1 macrophages and M2 macrophages, both of which are closely associated with tumour immunity [16]. Classically activated M1 macrophages are primarily involved in proinflammatory responses. Their activation is driven by factors such as lipopolysaccharide (LPS), interferon-γ (IFN-γ), granulocyte-macrophage colony-stimulating factor (GM-CSF), or other pathogen-associated molecular patterns [17–19]. Upon activation, proinflammatory factors, including IL-6, IL-12 and tumour necrosis factor (TNF), are produced. They possess the ability to identify and engulf tumour cells, thereby impeding tumour growth and metastasis. Furthermore, they can present tumour antigens to T cells, thus triggering specific immune responses and exerting antitumour effects [20–23]. M2 macrophages are macrophages that undergo alternative activation and primarily engage in anti-inflammatory responses; the activation of M2 macrophages is driven by macrophage colony-stimulating factor (M-CSF), IL-4, IL-10, IL-13, transforming growth factor-β (TGF-β), and glucocorticoids [16]. After polarization, cells can release anti-inflammatory cytokines (including IL-10, IL-13, IL-4, Arg-1, and CD206), thereby contributing to host defence, wound healing and tissue remodelling [24]. M2 macrophages can also promote tumour progression through various biological mechanisms, including the secretion of immunosuppressive molecules such as IL-10 and TGF-β, which hinder the function of other immune cells. Additionally, these cells secrete vascular endothelial growth factor (VEGF), which promotes tumour angiogenesis [25, 26].

TAMs are an important component of the TME, and they make up more than 50% of aggregate tumour cells [27]. The TME of solid tumours can recruit myeloid cells and lead to the extensive infiltration of immunosuppressive macrophages [28]. Macrophages in the TME are mainly derived from bone marrow-derived monocytes (BMDMs), which are recruited by tumour- or mechanism-derived chemokines [28–30]. Both M1 and M2 TAMs are present throughout all phases of tumour progression, with M1 macrophages prevailing during the initial stage and M2 macrophages dominating during the intermediate and advanced stages [31]. With tumour progression, M1 macrophages gradually polarise to M2 macrophages, and an increase in the quantity of M2 TAMs indicates a poor prognosis. Moreover, M2 macrophages promote tumour angiogenesis, leading to tumour progression; this role is the opposite of the role of M1 macrophages [32]. Therefore, decreasing the presence of M2 TAMs in the TME or fostering the conversion of M2 macrophages to M1 macrophages plays a significant role in the treatment of tumours [33].

Current research on macrophages in cancer immunotherapy is centred on unravelling their intricate functions within the tumour microenvironment and investigating strategies for translating this knowledge into clinical applications. A number of researchers have reported that engineered immune cells, which are an emerging form of immunotherapy, are immune cells that are engineered to recognise and respond to disease [34]. When these immune cells are introduced into patients, they can serve as a “living drug” that inhibits the growth of tumour cells [35]. Engineered macrophages, which are a subtype of modified immune cells, originate from macrophages and are primarily utilised for drug delivery, tissue repair, and antitumor applications through genetic engineering techniques [36–38]. Considering the abundance of macrophages in the TME, altering macrophages for solid tumour therapy has great potential. The techniques used to engineer macrophages and their immunotherapeutic impacts on the TME are the focus of this study.

### Common techniques for engineering macrophages

**Fig. 1:**
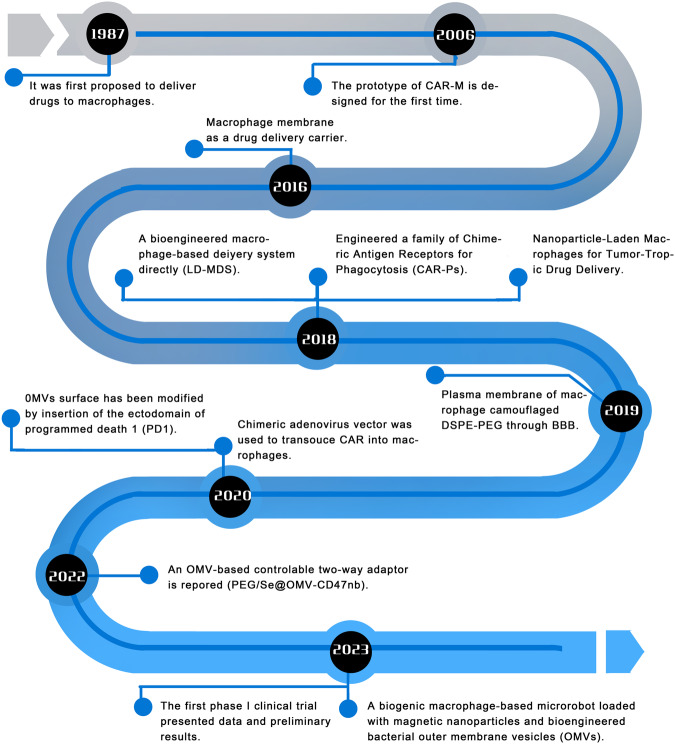
The evolution of engineered macrophages.

To date, there are three main methods for transforming macrophages into engineered macrophages. The process of developing engineered macrophages is shown in Fig. 1. First, macrophages were engineered for advanced drug delivery systems. However, the therapeutic effect is not optimal for solid tumours, mainly because the tumour has an immune barrier that reduces drug accessibility, which alters the therapeutic effect [39]. Macrophages, as an important part of the TME, possess the functions of natural immune cells and antigen-presenting cells and boast an extended half-life in blood; macrophages are essentially able to phagocytose foreign particles and specifically bind to tumour tissues [27, 40]. Therefore, macrophages can act as drug carriers and deliver drugs to tumour tissues. The first serious discussions and analyses of engineered macrophages for drug delivery emerged during the 1980s when micro particulates were proposed to function as a convenient platform for the targeted delivery of encapsulated drugs to cells within the mononuclear phagocyte series in vivo [41]. As research has progressed, macrophages were found to serve not only as direct or indirect carriers for drug delivery but also as encapsulate nanoparticles to facilitate drug delivery. Through these advancements, engineered macrophages have arisen as promising platforms for precision-targeted tumour therapy that are capable of overcoming immune barriers and drug delivery challenges encountered with traditional treatments [42, 43].

**Fig. 2:**
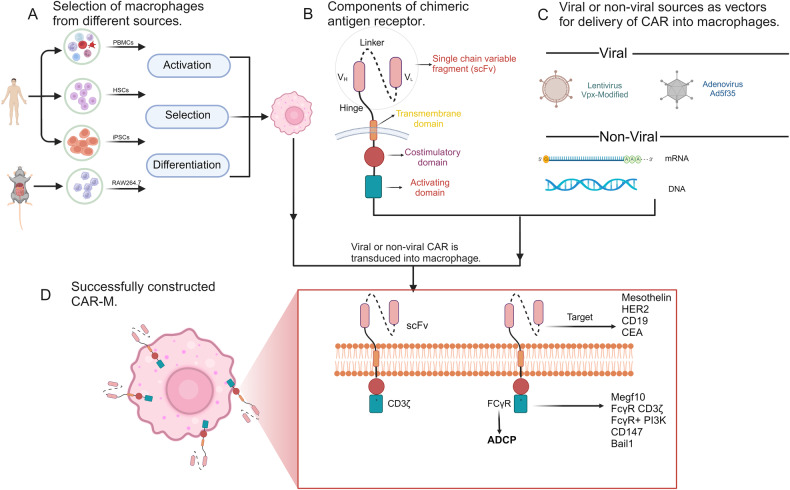
The structure of CAR-Ms. **A** Macrophages from different sources, such as BMDMs, monocytes derived from human peripheral blood mononuclear cells (PBMCs), and mouse-derived Raw264.7 cells, were selected. **B** CARs were composed of an intracellular domain, an extracellular domain and a transmembrane domain. **C** CARs were successfully transfected into viral or nonviral vectors of macrophages. **D** CAR-Ms were successfully constructed, and the diversity of the extracellular antigen and intracellular domain was determined. (created with biorender.com).

Chimeric antigen receptor-modified macrophages (CAR-Ms) have also been engineered. There has been tremendous progress in adoptive cell immunotherapy using CAR-T cells [44]. A challenging problem is the TME barrier formed by the extracellular matrix (ECM), which prevents CAR-T cells from infiltrating and persisting within the tumour immune microenvironment (TIME) [45–48]. A solution to this problem is the application of CAR-M therapy. The architecture of CARs in CAR-M therapy encompasses extracellular signalling domains that are designed to identify particular tumour antigens, hinge regions, transmembrane structural domains, and intracellular signalling structural domains (Fig. 2). Currently, scFv has been studied for use against common tumour targets, such as CD19 and HER2 [49–51]. Although the CAR-M intracellular domain does not contain immunoreceptor tyrosine-based activation motifs (ITAMs), it has CD3ζ chain-related functions [52, 53]. Macrophages can express the SH2 domain-containing kinase Syk, which binds to CD3ζ and transduces phagocytosis. Using chimeric antigen receptor technology, engineered macrophages (CAR-Ms) offer a promising approach for targeted therapy against specific tumour antigens. This innovative strategy provides not only a more precise and efficient treatment option for cancer patients but also the ability to surmount the constraints that have hindered the use of conventional CAR-T-cell therapy [54, 55].

The third is the application of bacterial outer membrane vesicles (OMVs) in engineered macrophages. OMVs, which were first thought to be the product of the disordered growth of gram-negative bacteria, are naturally occurring nanoparticles that are secreted by gram-negative bacteria with a particle size of 30-100 nm [56]. OMVs consist of antigens that originate from bacteria and various intrinsic pathogen-associated molecular patterns (PAMPs), which are highly immunogenic and, therefore can be used as bacterial vaccines and adjuvants to activate both humoral and cellular immune responses [57–59]. As vaccine carriers, OMV has more reliable safety performance than bacteria. Currently, there are various means for the synthetic modification of OMVs [60]. The first is by constructing different tumour antigen types immobilised on the surface of OMVs, such as Spy C and L7Ae [61, 62]. The second is to carry a PD-L1/CD47 nanoantibody that can be delivered to the TME to promote tumour-associated macrophage remodelling to an antitumor state [63, 64]. The third is to modify OMVs by synthetic methods to help maintain their stability in the human body and artificially control the release of antigens or antibodies, which plays a role in precise treatment [62, 65].

The aforementioned three methods for the engineered modification of macrophages represent the primary research areas in the current landscape. In the subsequent sections, I shall furnish a comprehensive exposition and delve into meticulous discussions regarding the advancement and utilisation of these engineering techniques.

### Progress in the application of engineered macrophages in tumour immunotherapy

Over the past two decades, a multitude of scholars have endeavoured to ascertain whether engineered macrophages can play a role in boosting immunotherapy in cancer. Indeed, they demonstrated the role of engineered macrophages in enhancing the efficacy of immunotherapy. There is no doubt that engineered macrophages have broad prospects in future tumour immunotherapy [17, 34, 35, 39]. As a burgeoning form of immune cell therapy, engineered macrophages not only mitigate the immune system’s exposure to drug-related injury but also augment the immune response and fortify the efficacy of immunotherapy.

### Engineered macrophages for advanced drug delivery systems

**Fig. 3:**
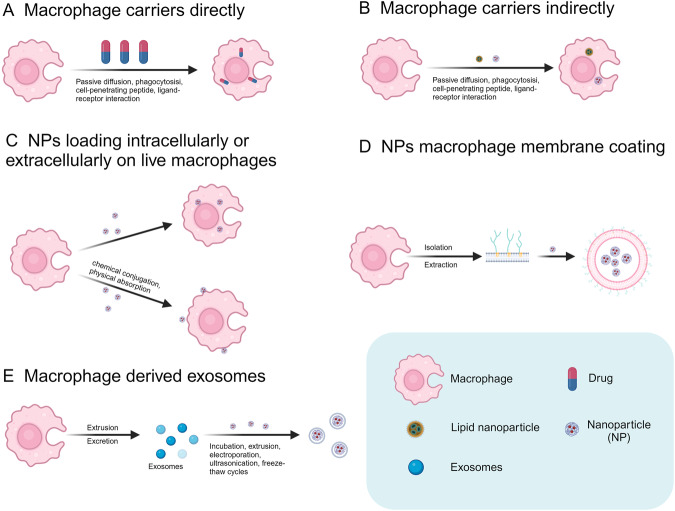
The various ways engineered macrophages deliver drugs. **A** Macrophages can be direct carriers for the transportation of anticancer drugs, and **B** macrophages can be indirect carriers for the transportation of anticancer drugs. **C** Nanomedicines can be loaded intracellularly or extracellularly on live macrophages, and **D** nanomedicines can be encapsulated by macrophage membranes. **E** Nanomedicine loading in macrophage exosomes. (created with biorender.com).

In this segment, three methodologies for employing engineered macrophages in drug delivery shall be elucidated (Fig. 3): 1) the use of engineered macrophages as direct drug carriers; 2) the use of engineered macrophages as indirect drug carriers; and 3) the use of engineered macrophage-encapsulated nanoparticles as drug carriers.

#### Engineered macrophages as direct drug carriers

There exist two primary reservoirs of M1 macrophage drug carriers, bone marrow-derived macrophages and RAW264.7 macrophages, which are capable of binding to cancer cells through their own α4 and β1 integrins and interacting with VCAM-1 [66]. Studies conducted earlier have demonstrated the loading of doxorubicin (DOX) into a mouse macrophage-like cell line (RAW264.7), which culminated in the establishment of a biomimetic drug delivery system (BDS). The generated BDS successfully targeted tumour cells and did not diminish their tumorigenic potential when treating metastatic 4T1 mouse mammary carcinoma cells. Furthermore, the BDS substantially extended the lifespan of mice [64, 67]. In a study by Huang et al., engineered macrophages (Oxa(IV)@ZnPc@M) was developed to carry nanodrugs consisting of oxaliplatin prodrugs and photosensitizers. These macrophages functioned as near-infra-red light-triggered drug carriers that are capable of inducing M1-type polarisation in vivo [68]. Oxa(IV)@ZnPc@M exhibited adept penetration into both primary and bone-metastatic tumours to achieve light-controlled drug release and chemo-photodynamic combination therapy, which not only provides precise drug release and improves the efficiency of drug administration but also induces immunogenic cellular death and plays an antitumour role. Within a specific dosage range, anticancer medications, including 5-fluorouracil, irinotecan, cisplatin, and gemcitabine, which can be combined with chemotherapy, radiotherapy, and immunotherapy, did not markedly impact the viability of macrophages [69].

In conclusion, this strategy not only enhances the local drug concentration and reduces systemic side effects but also assists in surmounting the immune barrier within the tumour microenvironment, thus revealing a novel strategy for addressing ailments such as cancer [70].

#### Engineered macrophages as indirect drug carriers

In contrast, when macrophages serve as indirect conveyors of anticancer medications, they not only bypass the effects of drugs on the macrophages but also increase the drug-loading ratios [71]. Engineered macrophages mainly transport liposomes or nanoparticles loaded with anticancer drugs. The present findings confirmed that drugs loaded via nanoparticles are more efficient than those carried directly [56, 72]. Recently, investigators have examined the effects of M1 macrophages instead of macrophage-loaded drugs in the treatment of glioma. First, poly (lactide-coglycolide) nanoparticles were combined with primary M1 macrophages to create M1 macrophage-loaded nanoparticles [73]. Second, in vitro cellular assays indicated that M1 macrophages not only exhibited formidable tumour-suppressing effects but also facilitated the transport of antineoplastic agents across the blood-brain barrier into tumour tissues [74, 75]. Finally, M1 macrophage-loaded nanoparticles seemed to improve the targeting efficiency and efficacy of chemotherapeutic drugs in glioma treatment. Similarly, the development of hybrid exosomes through the fusion of macrophage extracellular vesicles with synthetic liposomes represents a novel approach for drug delivery in cancer therapy. These hybrid exosomes have the capability to encapsulate chemotherapeutic agents, thereby increasing their cytotoxicity towards cancer cells. Moreover, these exosomes exhibit pH-sensitive drug release, particularly within acidic tumour environments, thus facilitating the precise delivery of therapeutic agents to cancer cells. This innovative approach has immense potential for augmenting the efficacy and precision of cancer therapeutics [76].

The findings suggested that macrophages, as indirect carriers of drugs, possess the capability to traverse the blood-brain barrier not only in gliomas but also in some other solid tumours through the immunosuppressive microenvironment. This innovation not only enhances the precise targeting and therapeutic efficacy of drugs against tumours but also optimises drug potency, thereby significantly enhancing the overall response to cancer immunotherapy [73, 77–81].

#### Engineered macrophage-encapsulated nanoparticles as drug carriers

Currently, there are three main methods of engineering macrophage-encapsulated nanoparticles (Fig. 3): nanomedicines are loaded inside or on the surface of living macrophages; nanomedicines are encapsulated by macrophage membranes; and nanomedicines are loaded in macrophage exosomes. The macrophage membrane surface abounds with lipids and proteins, thus presenting an opportunity for loading nanodrugs through chemical conjugation or physical adsorption [72]. The drug loading method can effectively prolong the drug circulation time, specifically by targeting deeper regions of the tumour and augmenting the effectiveness of the anticancer medication [72, 82]. In a recent study, macrophage-encapsulated mesoporous silica cocoon materials were developed to deliver DOX to 4T1 tumours [83]. Macrophage membrane-encapsulated mesoporous silica cocoon materials were able to effectively prolong the survival of nanoparticles in the blood circulatory system and increase their accumulation in tumours, suggesting that macrophage membranes can act as attachment sites for nanomedicines to target tumours and improve therapeutic efficacy.

Macrophage membrane drug delivery is principally segmented into four steps: macrophage isolation, purification, the preparation of nanocarrier cores and fusion of the “core-shell” structure. This drug delivery method disrupts the structure of macrophages and uses cell membranes to encapsulate nanomedicines [84]. A more recent study using a drug delivery system composed of nanogemcitabine encapsulated by a macrophage membrane showed that the drug delivery system promoted lymphocyte infiltration into the tumour with effective intratumor infiltration and drug release ability, thereby significantly removing multifunctional immunosuppressive cells [85]. Furthermore, this system enhances lymphocyte infiltration into tumour regions and, in combination with anti-PD-L1 drugs, effectively decreases the number of nonfunctional cells; thus, this system showed encouraging outcomes in various tumour models.

Compared with synthetic nanoparticles, the use of exosomes as nanocarriers for targeted drug delivery has many advantages [86]. Macrophage-derived exosome vesicles can be used as carriers of anticancer drugs; these vesicles can maintain the same favourable surface properties as macrophage membranes, enter the body and fuse with the membrane for cellular uptake [87]. To address the impact of the blood-brain barrier, as well as the hypoxic microenvironment, on the treatment of glioblastoma, Wu et al. generated a silica nanoparticle encapsulating catalase (CAT@SiO2) that adheres to exosomes derived from macrophages, thus forming CSI@Ex-A, which exhibits remarkable blood-brain barrier penetration and adept targeting of cancer cells [88]. CAT released by tumour cells after the endocytosis of CSI@Ex-A catalyses the decomposition of hydrogen peroxide, thereby generating oxygen to mitigate tumour hypoxia. More generally, these basic findings are consistent with research showing that the drugs carried by exosomes have good biocompatibility and long cycle times while improving the drug loading rate and safety [76, 88].

Macrophages can serve as drug carriers to target diseased tissues by leveraging their inherent migratory properties to deliver therapeutic payloads precisely to target sites. This approach capitalises on the extended half-life of macrophages in blood and their responsiveness to the pathological microenvironment, thereby minimising drug distribution in healthy tissues and associated side effects. Although this method has yet to be implemented clinically, future research endeavours will delve into optimising the design of macrophages as drug delivery vehicles and overcoming the challenges associated with clinical translation. The goal of these efforts is the clinical application of this promising strategy in treating various diseases [39, 89, 90].

### Chimeric antigen receptor-modified engineered macrophages

**Fig. 4:**
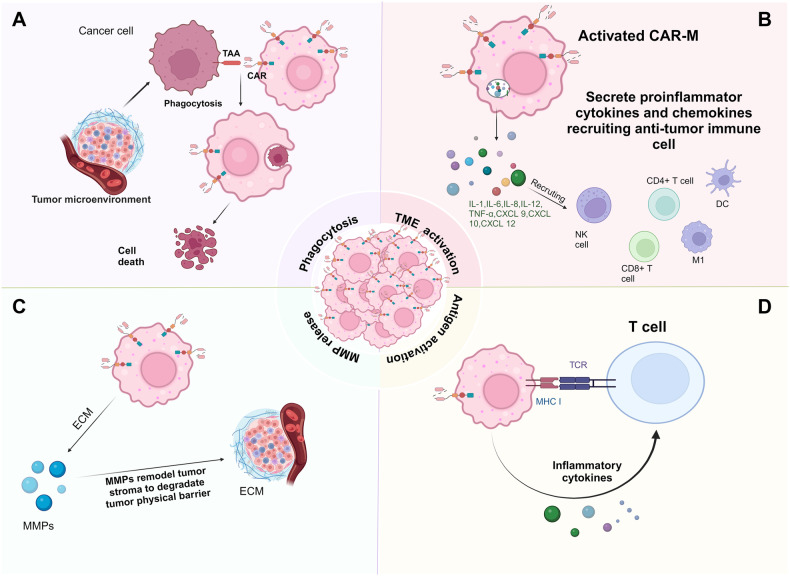
Major mechanisms of solid tumour clearance by CAR-Ms. **A** CAR-Ms engulf and kill tumour cells by recognising tumour cell-associated antigens. **B** Activated CAR-Ms can secrete proinflammatory factors and chemokines to recruit antitumour immune cells to tumour tissues. **C** CAR-Ms can induce the secretion of matrix metalloproteinases to degrade dense tumour ECM. **D** CAR-Ms can coordinate an antitumour T-cell response by recruiting T cells and cross-presenting antigens from phagocytosed cells. (created with biorender.com).

Numerous endeavours have sought to employ CAR-Ms for cancer treatment (Table 1). Figure 4 illustrates the mechanism by which chimeric antigen receptor macrophages target solid tumours. During 2006, the initial in-depth discussions and analyses of CAR-Ms began with the fusion of a single-chain Fv molecule targeting human carcinoembryonic antigen (CEA) with the transmembrane and cell membrane structural domains of human CD64. This construct was transferred into monocytes using adenoviral vectors and chimerized within the cell membrane [91]. These results clearly show that CAR-M effectively delays the growth of CEA-positive tumour cells in vitro.

**Table 1:** All studies of CAR-M-based therapies to date, as well as the composition, macrophage source, and mode of CAR-M delivery.

Macrophage source	Gene delivery	Target antigens	Extracellular/ Intracellular domains	Application strategy	Clinical trials	Refs.
Monocytes derived from human PBMCs	Adenoviral vector	CEA	Extra:scFv Intra: CD64	In vitro	/	[91]
PBMCs	MaxCyte GT System	Mesothelin	Extra: scFv Intra:CD3ζ	In vitro	NCT03608618	[50]
J774A.I Macrophages BMDMs	Lentiviral vector	CD19 CD22	Extra: scFv Intra: Megf10, FcγR CD3ζ, FcγR + PI3K	In vitro	/	[117]
Raw264.7	Lentiviral vector	HER2	Extra: scFv Intra: CD147	In vitro	/	[95]
THP-1 monocyte-derived macrophages	Adenoviral vector(Ad5f35)	CD19 HER2	Extra: scFv Intra: CD3ζ	In vitro	NCT04660929	[28]
iPSCs from PBMCs	Lentiviral vector	CD19 Mesothelin	Extra: scFv Intra: CD86-FcγR I	In vitro	/	[118]
PBMC	Ad5f35	HER2	Extra: scFv Intra: CD3ζ	In vitro	/	[119]
RAW264.7, M2 BMDMs, M2 TAMs	Non-viral vector: jetPEI-macrophage(MPEI)	CD19 ALK	Extra: scFv Intra: CD28-CD3	In vivo	/	[120]
Raw264.7 monocyte	Lentiviral vector	CCR7	Extra:CCL19 Intra:TLR2,TLR4,TLR6,MerTK,4-1BB-CD3ζ	In vitro	/	[121]
THP-1 BMDMs RAW 264.7	nanoporter(NP)-hydrogel	CD133	Extra: scFv Intra: CD3ζ	In vivo	/	[51]
THP-1 hematopoietic stem and progenitor cells (HSPCs)	Lentiviral vector	CEA	Extra: scFv Intra: CD28-CD3ζ	In vitro	/	[122]
RAW264.7 BMDMs	Lipid nanoparticle (LNP)	CD19	Extra: scFv Intra: CD3ζ	In vitro	/	[123]
Human primary peritoneal macrophages	Lentiviral vector	HER2	Extra: scFv Intra: FcεR1γ	In vitro, In vivo	/	[124]
BMDMs	Plasmids	ErbB2	Extra: scFv Intra: CD3ζ	In vitro, In vivo	/	[125]

CAR-Ms were first demonstrated to exert effective antitumour effects in vivo in a recent study by Klichinsky et al., who used the targeting of the solid tumour antigen mesothelin or HER2 as the antigenic structural recognition domain and CD3ζ as the intracellular structural domain [28]. Concurrently, they engineered a minimally replicating chimeric adenoviral vector (Ad5f35) for macrophage-mediated CAR delivery [92]. The findings indicated that Ad5f35-transformed human macrophages exhibited robust CAR expression, leading to dose- and time-dependent elimination of the HER2+ ovarian cancer cell line SKOV3 by CAR-Ms. The extent of tumour phagocytosis and cytotoxicity exhibited a direct correlation with both the magnitude of CAR expression and the level of target antigen expression. Moreover, HER2-CAR-Ms demonstrated the ability to polarise M2 macrophages into M1 macrophages, incite the formation of an inflammatory TME, and increase T cell cytotoxicity against tumours.

One pivotal factor contributing to the inefficacy of CAR-T therapy in solid tumours lies in the challenge of T-cell infiltration into tumour tissues, which are enriched with dense and distinctive ECM [93]. The compact tissue structure of tumour tissues constitutes a physical impediment that constrains the migration of T cells. Matrix metalloproteinases (MMPs) primarily regulate the synthesis and degradation of the ECM [94]. In response to this constraint, efforts to formulate CAR-147-M, which is composed of a monoclonal antibody in a single-chain format that specifically targets human HER2 and encompasses the hinge region of IghG1, were made. Additionally, CAR-147-M incorporates the transmembrane and intracellular domains derived from mouse CD147 molecules [95]. These findings indicated that CAR-147 macrophages attenuated tumour collagen deposition and facilitated T-cell infiltration into tumours; however, in vitro, CAR-147 macrophages did not impede tumour cell proliferation.

The FDA has granted approval to nine CAR-T-cell therapies for haematologic malignancies (Table 2) [44]. Two clinical trials based on CAR-M therapeutic interventions are already available (NCT05007379 and NCT04660929). The initial one is the drug candidate CT-0508 from Carisma Therapeutics, which is tailored to patients with relapsed/refractory HER2 overexpressing tumours (Phase I trial). This study enrolled 18 patients with HER2-positive solid tumours for an inaugural investigation into the efficacy of CAR-M transduction via adenovirus. Carisma Therapeutics unveiled data and preliminary findings from a phase I clinical trial of CT-0508 catering to individuals afflicted with HER2-positive solid tumours, as highlighted at the American Society for Gene and Cell Therapy conference in 2023, with a particular emphasis on its safety, tolerability, efficacy in cell production, transportation, and modulation of the tumour microenvironment [48]. Another notable finding is that Maxyte’s MCY-M11 leverages mRNA-transfected PBMCs to express mesothelin-targeting CARs, including CAR-Ms, as a therapeutic intervention for patients with recurrent or resistant cases of ovarian cancer and peritoneal mesothelioma. Nevertheless, there is an ongoing recruitment of volunteers for the phase I clinical trials [50]. In addition, a great quantity of other clinical trials related to CAR-M therapy are underway (NCT06224738, NCT04405778 and NCT05164666). The further development of CAR-M therapy will require strong evidence from clinical trials.

**Table 2:** Nine marketed CAR-T therapeutic drugs.

Drug name	Target	Indications	Complete remission rates	Approval time	Listed country
Kymriah	CD19	Relapsed or refractory diffuse large B-cell lymphoma; B cell precursor acute lymphoblastic leukaemia	>90%	2017.8	America
Yescarta	CD19	Relapsed or refractory diffuse large B-cell lymphoma; Recurrent or refractory follicular cell lymphoma	51%	2017.10	America
Tescarta	CD19	Recurrent or refractory mantle cell lymphoma	67%	2020.7	America
Breyanz	CD19	Relapsed or refractory diffuse large B-cell lymphoma	54%	2021.2	America
Abecma	BCMA	Relapsed or refractory multiple myeloma	28%	2021.3	America
Axicabtagene Ciloleucel	CD19	Relapsed or refractory diffuse large B-cell lymphoma	/	2021.6	China
Relmacabtagene Autoleucel	CD19	Relapsed or refractory diffuse large B-cell lymphoma	/	2021.9	China
Carvykti	BCMA	Relapsed or refractory multiple myeloma	78%	2022.2	America
Relmacabtagene Autoleucel	BCMA	Relapsed or refractory multiple myeloma	/	2023.6	China

### Application of bacterial outer membrane vesicles in engineered macrophages

**Fig. 5:**
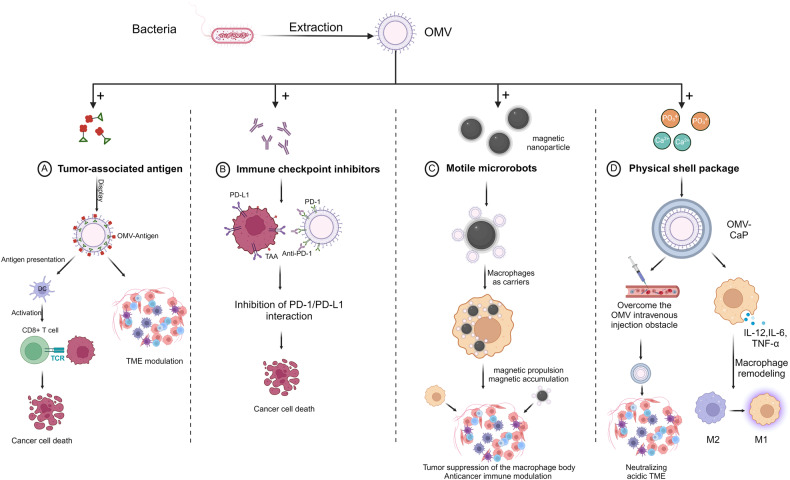
The main mechanism by which bacterial outer membrane vesicles are modified to treat solid tumours. **A** Tumour-associated antigens carried by OMVs activate T cells to induce the immune system to kill cancer cells in vivo. **B** Immune checkpoint inhibitors carried by OMVs can block immunosuppression. **C** Macrophages carrying magnetically driven OMVs can accurately target tumour tissues. **D** Physically encapsulated OMVs can overcome intravenous obstruction and induce the conversion of M2 macrophages to M1 macrophages. (created with biorender.com).

Regulating and reprogramming macrophages by constructing OMVs that target the TME is crucial for immunotherapy [33]. In this section, two strategies by which OMVs synergistically regulate macrophages in the TME are discussed: (1) OMVs directly regulate engineered macrophages, and (2) OMVs indirectly regulate engineered macrophages (Fig. 5).

### OMV directly regulate engineered macrophages

Past investigations have documented the utilisation of a calcium phosphate shell to encase OMVs [96]. CaP not only overcomes the obstacles of antibody-dependent clearance and high toxicity induced by OMVs when administered intravenously, but CaP is also sensitive to an acidic TME and can promote neutralisation of the acidic TME. In vivo, injection of the modified OMV-CaPs precipitated the transition of tumour-associated macrophages from the M2 to the M1 state, which improved the therapeutic efficacy of tumour treatment. OMV-CaP administration also increased the level of secreted cytokines (IL-12, IL-6, TNF-α, and IFN-γ) in tumour tissues (e.g., breast cancer) and promoted an antitumour immune response. This finding suggested that OMVs have the powerful ability to remodel the TME.

Owing to the diminished oxygen levels within the TME, tumour cells under hypoxic circumstances can facilitate metastasis by suppressing glycolysis in macrophages, thus reducing their competition for glucose with neovascularized epithelial cells [97]. Therefore, in a previous study, researchers modified OMVs for macrophages by synthesising REDD1-siRNA with the pH-sensitive linkers cis aconitum anhydride and paclitaxel [98]. The findings revealed a decrease in the mRNA expression of Redd1 during in vitro experiments, and the phagocytic capacity and number of macrophages polarised from the M2 to M1 phenotype increased. The results of the study clearly demonstrated that OMVs can reprogram macrophages to enhance the efficacy of tumour immunotherapy [99].

The interplay between CD47 and signal-regulated protein α (SIRPα) initiates an inhibitory signalling pathway, which leads to malignant cell evasion of macrophage phagocytosis [100]. OMV-CD47nb, a bidirectional adapter, can simultaneously interact with CD47 on tumour cells and Toll-like receptors (TLRs) on TAMs and block “do not eat me” signalling on tumour cells while polarising TAMs to the M1 phenotype. The outermost PEG/Se layer additionally mitigated the immunogenicity and toxicity of intravenously injected OMVs. Conversely, localised irradiation at the tumour site disrupts the PEG/Se layer, resulting in a more potent and targeted release of OMVs into the TME [101, 102]. This finding suggests that OMVs can improve treatment efficacy when combined with immune checkpoint inhibitor therapy [62].

### OMVs indirectly regulate engineered macrophages

Cancer immunotherapy can block immunosuppressive TME through immune checkpoint inhibitors, such as anti-PD 1/PD-L1 therapy [103]. OMVs that express PD1 bind specifically to tumour cell membranes, resulting in PD-L1 internalisation and disruption of PD-L1-mediated immunosuppression, thereby promoting T-cell proliferation [104]. Recent research suggested that an OMV-based mRNA delivery platform (OMV-LL) was constructed by combining an RNA-binding protein (L7Ae) and a lysosomal escape protein (*Listeria monocytogenes* lysin O); this strategy emulates the innate generation of tumour antigens within malignant cells [105]. These findings demonstrated the ability to provoke a vigorous, antigen-specific immune response and enduring immunity. This study provides a “plug-and-play” strategy to implement mRNA antigens in an OMV-based platform, which could be widely applied for personalised tumour vaccines [106].

Recent studies using magnetically driven immune macrophage microrobot (MΦ-OMV) analysis have suggested that controlling MΦ-OMV by magnetic operation can target tumour site aggregation and play a role in precise guidance [107]. Importantly, MΦ-OMV contains two antitumour peptides (hirudin and mastoparan 1), which inhibit tumour angiogenesis and promote cancer cell apoptosis, respectively. Moreover, tumours can be eliminated through a variety of mechanisms [108, 109]. This design methodology holds immense promise for fabricating dynamic medical vectors that meet the rigorous criteria of clinical trials, including commendable biocompatibility with minimal adverse effects, controlled propulsion, tumour targeting, and multimodal therapies, which are promising for the future of medicine. Furthermore, gold nanoparticles combined with OMVs to generate Au-OMVs, when combined with radiotherapy, were able to induce macrophage chemotaxis towards glioma cells [106, 110, 111]. In a recent study, a multifunctional nanosystem (mU@OMVs) was constructed based on OMVs. This system blocks efferocytosis, which acts on M2 macrophages to reduce the phagocytosis of apoptotic cells [112]. Recent evidence suggests that bacterial-derived OMVs possess the dual functions of carriers and adjuvants, so they are more suitable as carriers for tumour vaccines than synthetic nanoparticles [106].

In conclusion, OMVs play a pivotal role in engineered macrophages by activating their immune response through the transport of microbial-related molecular patterns, thereby enhancing their phagocytic capabilities, facilitating antigen presentation, and potentially expanding macrophages to target cancer cells by presenting specific antigens or expressing tumour-related peptides. Furthermore, OMVs, which serve as nanoscale carriers, can be utilized for drug delivery by transporting therapeutic agents directly into the tumour microenvironment, thereby contributing to cancer treatment. Currently, this strategy remains in the research phase but holds significant promise for future clinical applications.

## Conclusions and future outlook

As mentioned above, significant advancements have been achieved in the engineering of macrophages for cancer immunotherapy. The ability of these “living drugs” to precisely target the TME, as well as solid tumours, has ameliorated the shortcomings of cancer immunotherapy in solid tumours, but there are still many shortcomings and challenges with this novel therapy.

First, in terms of modifying macrophage drug delivery, the mechanism governing the recruitment and polarisation of macrophages in the TME remains elusive [40, 113, 114]. Similarly, due to the complex nature of the TME, macrophage-mediated drug delivery may not be sufficient to eliminate tumours. In terms of safety, engineered macrophages may elicit unpredictable immune responses, potentially resulting in immune system overactivation and leading to inflammation, autoimmune diseases, and other unforeseen risks. Regarding efficacy, the behaviour of engineered macrophages in vivo can vary among individuals, leading to significant differences in their antitumor effects across different patients. Addressing the challenge of controlling variations in efficacy among individuals poses a significant hurdle. In the future, if this therapy can be combined with immune checkpoint blockade (ICD) therapy, phototherapy and other therapies, such developments could further augment the efficacy of tumour treatment. Second, in terms of CAR-M therapy, the technology is still immature. Although clinical trials of this immunotherapy are available, the results have not been announced. An additional groundbreaking discovery revealed that the TME has the capacity to divert tumour-resident CAR-Ms towards a phenotype that supports tumour growth, in contrast to preclinical modelling results (CAR-Ms reprogramme the TME) [115]. Because of this potential limitation, it is imperative for us to delve into the mechanism underlying this phenomenon. Third, OMVs synergise with macrophages to alter the TME. The utilisation of the OMV-based adapter strategy extends to therapeutic approaches involving other immune checkpoints, such as the CD24-Siglec-10 axis [62]. The current limitations are that this strategy is technically difficult to perform, and quantification and timeliness are difficult to ensure. Future studies designed to answer these questions will certainly help increase OMV applicability and design diversity in the field of tumour vaccines. Fourth, in the realm of clinical translation, the development and production costs associated with engineered macrophage therapies are typically substantial, thus necessitating stringent patient requirements. Addressing cost considerations and managing patient relationships in clinical applications are of paramount importance. In conclusion, engineered macrophages, as an emerging cancer treatment modality, present both potential and challenges [116].

In the face of these obstacles, macrophages have the potential to emerge as a potent tool for the treatment of solid tumours. Future progress in cancer immunotherapy will require collaborative interdisciplinary endeavours, with combination therapies harnessing the collective knowledge of proteins, chemistry, materials, and bioengineering to revolutionise immune cell therapies for individuals battling cancer.

## Data Availability

All the data supporting the findings of this study are available from the corresponding author upon reasonable request.
